# Influence of Red Seaweed Polysaccharides on Gel Properties and *In Vitro* Antioxidants of Surimi Product Fish Balls

**DOI:** 10.3390/foods15061018

**Published:** 2026-03-13

**Authors:** Menghan Ma, Tao Hong, Zhipeng Li, Yanbing Zhu, Yuanfan Yang, Hui Ni, Zedong Jiang, Mingjing Zheng

**Affiliations:** 1 College of Ocean Food and Biological Engineering, Jimei University, Xiamen 361021, China; menghanma1108@163.com (M.M.); taohong94@jmu.edu.cn (T.H.); lzp2019@jmu.edu.cn (Z.L.); yanbingzhu@jmu.edu.cn (Y.Z.); yuanfan@jmu.edu.cn (Y.Y.); nihui@jmu.edu.cn (H.N.); 2 Fujian Provincial Key Laboratory of Food Microbiology and Enzyme Engineering, Xiamen 361021, China; 3 Xiamen Ocean Vocational College, Xiamen 361021, China

**Keywords:** fish ball, red seaweed polysaccharide, gel properties, antioxidant activity, *in vitro* digestion

## Abstract

The effects of red seaweed polysaccharides, e.g., carrageenan, agar gum, *Porphyra haitanensis* polysaccharide (PHP), and *Bangia fusco-purpurea* polysaccharide (BFP), on the physicochemical properties and *in vitro* antioxidants of silver carp surimi gels were studied. Adding appropriate concentrations of carrageenan and agar gum increased hydrophobic interactions, resulting in a denser and more uniform gel network as observed by SEM, and shortened the relaxation time of the fish balls, thus improving the gel strength and hardness of the products. When adding 0.75% carrageenan and 0.50% agar gum, the gel strength of the fish balls reached its maximum value, increasing by approximately 28.84% and 12.08%, respectively, compared to the control group (*p* < 0.05). However, with the over-addition of PHP and BFP, the cross-linking of surimi proteins was inhibited, resulting in a decrease in gel strength and hardness. In addition, red seaweed polysaccharide improved the free radical scavenging activity of fish balls, especially fish balls with 0.50% and 1.00% PHP and BFP exhibited better free radical scavenging activity after digestion. These findings offer insights and actionable strategies for enhancing the gel properties and function of surimi products with seaweed polysaccharides.

## 1. Introduction

Surimi, a concentrated myofibrillar protein from fish, undergoes a process of deboning, chopping, and washing to eliminate impurities such as myoplasmic proteins, lipids, and blood components [1]. The gelatinous properties of surimi make it an ideal raw material for products like fish balls. These gelatinous foods have high market acceptance due to their delicate texture, excellent nutritional profile, and price advantage. Silver carp, a common freshwater aquaculture species in China, is frequently processed into various surimi products [2]. As previously reported, freshwater fish surimi exhibits relatively poor gel strength, primarily attributed to its lower content of amino acid and insoluble myofibrillar proteins, when compared to its mammalian meat surimi counterpart [3]. Silver carp is one such freshwater fish whose surimi is extremely difficult to gelatinize and highly susceptible to gel deterioration. This not only leads to an undesirable taste but also restricts the processing and utilization of silver carp surimi. Thus, research efforts have been dedicated to enhancing surimi gel quality by either optimizing processing parameters [4] or by incorporating exogenous additives, such as hydrocolloids [1], polyphenols [5], and enzymes [6].

Polysaccharides are widely distributed, abundant natural biopolymers. Most of them exhibit prominent rheological properties, including thickening, stabilizing, and gelling capabilities. Owing to their non-toxicity, extensive availability, and renewability, polysaccharides are being increasingly utilized in food processing to enhance food quality. Studies have demonstrated that composite gels formed by proteins and polysaccharides typically possess superior gel-forming capacity and more stable gel structures compared to single-protein gel systems [7]. Specifically, red seaweed polysaccharides like carrageenan and agar gum are commonly incorporated into surimi products as texture modifiers to enhance quality [1,8]. Carrageenan, agar gum, *Porphyra haitanensis* polysaccharide (PHP), and *Bangia fusco-purpurea* polysaccharide (BFP) are common red seaweed polysaccharides. Carrageenan and agar gum are both hydrophilic colloids with gelling properties. By exerting thickening, gelling, and stabilizing effects, they can regulate the rheological characteristics of food systems [9,10]. Compared to carrageenan and agar gum, PHP and BFP exhibit poor gel-forming ability and are primarily utilized as functional ingredients in food systems. These differences stem from their distinct structural properties, which lead to varying interactions with proteins and consequently alter the quality of the composite gel. Research has indicated that surimi gels exhibit improved strength and texture when carrageenan is added at levels of 1–3 g/kg [1]. Cao et al. have reported that carrageenan concentrations of 0.1%–0.4% can enhance the structural properties of frankfurters, promoting the formation of a tighter network structure [11]. Similarly, Zheng et al. [8] have discovered that the gel strength and textural characteristics of surimi products are unaffected by the addition of 0.125% and 0.25% agar gum. Furthermore, developing novel surimi products that combine both gel-enhancing and health-promoting functions has become a research hotspot. Such products not only pursue superior textural characteristics but also emphasize imparting additional health benefits through the incorporation of functional additives. Our previous study has found that the seaweed polysaccharide fucoidan is capable of enhancing the antioxidant activity of surimi products [12]. However, research on how seaweed polysaccharides from different sources affect the functional properties and nutritional value of surimi products remains limited. Existing studies primarily focus on the effects of individual polysaccharides, with few comparative analyses examining the impact of multiple polysaccharides within the surimi system.

Therefore, in this study, the silver carp surimi gel products, i.e., fish balls, were prepared with different red seaweed polysaccharides, namely carrageenan, agar gum, PHP, and BFP. The effects of different red seaweed polysaccharides on the gel properties and nutritional value of surimi products were investigated by determining the gel strength, textural property analysis, water distribution, microstructure, and antioxidant capacity of fish balls. The results of this study introduce new possibilities for the high-value use of red seaweed polysaccharides in the food industry and theoretically support the creation of functional surimi products incorporating polysaccharides.

## 2. Materials and Methods

### 2.1. Materials and Chemicals

Silver carp surimi was obtained from Fujian Anjoy Foods Co. Ltd. (Xiamen, Fujian, China). *Porphyra haitanensis* and *Bangia fusco-purpurea* were obtained from Fishery Taste Aquatic Products Co. Ltd. (Zhangzhou, Fujian, China). Carrageenan (LV-R-03, purity 99%, food grade) and agar gum (QI00, purity 99%, food grade) were purchased from Greenfresh Food Co. Ltd. (Zhangzhou, Fujian, China). Tapioca starch, phosphate, and salt were commercially available.

### 2.2. Extraction of PHP and BFP

Polysaccharides were prepared using the water extraction technique [13]. Distilled water (2 L) was combined with fifty grams of dried *Porphyra haitanensis* and *Bangia fusco-purpurea*, the mixture was heated to 90 °C for 2 h, and this procedure was repeated twice. The original liquid was concentrated using a rotary evaporator set at 45 °C. To the concentrated solution was added four volumes of 95% ethanol, after which it was maintained at 4 °C for 24 h. Centrifugation at 8085× *g* for 20 min was used to obtain the precipitated crude polysaccharides. Proteins were removed from crude polysaccharides using the Sevage method. The crude polysaccharide solution was combined with Sevage reagent (chloroform: n-butanol = 4:1, *v*/*v*) at a 1:2 ratio (*v*/*v*), followed by vigorous shaking and centrifugation at 8085× *g* for 15 min. The denatured proteins were discarded, and the aqueous phase was collected. This procedure was repeated three times. PHP and BFP were obtained by lyophilizing the polysaccharide solution at −55 °C and 20 Pa for 48 h following dialysis in a 3500 Da membrane for 72 h.

### 2.3. Preparation of Fish Balls

As reported previously [8], 1350 g of thawed silver carp surimi was weighed out and minced for 2 min. After adding 10 g of salt, the mixture was diced for a further 8 min. Next, 50 g of starch, 0.5 g of transglutaminase, 1 g of complex phosphate (sodium pyrophosphate: sodium hexametaphosphate: sodium tripolyphosphate = 1:1:1, which is used to improve the water-holding capacity and gel properties of surimi gels), 80 g of ice water, and red seaweed polysaccharides (in dry powder form) at various concentration levels (0%, 0.25%, 0.50%, 0.75%, 1.00%, and 1.25% *w*/*w*) were incorporated, followed by a final chopping step for 8 min. The fish balls were subsequently molded into round pellets then heated at 45 °C for 30 min, followed by 90 °C for 10 min. Following heating, the fish balls were packed and kept at −20 °C after being submerged in ice water for 20 min. The materials were thawed and used for further tests after being frozen for seven days.

### 2.4. Gel Strength Determination of Fish Balls

As reported previously [14], the gel strength of fish balls of 20 × 20 × 20 mm was determined using a TA.Touch texture analyzer (Shanghai Baosheng Industrial Development Co. Ltd., Shanghai, China). The probe model employed was P/5S, and the test was conducted in deformation mode with the following parameters: pre-test speed of 2 mm/s, test speed of 1 mm/s, post-test speed of 2 mm/s, and a trigger force of 5 g. The gel strength was calculated according to the following formula:

(1) Gel strenght(g×cm)=F×D

where F represents breaking force, g; D represents the deformation, cm.

### 2.5. Textural Property Analysis (TPA) of Fish Balls

As reported previously [15], the textural parameters (hardness, chewiness, and springiness) of 20 × 20 × 20 mm fish balls containing graded concentrations of red seaweed polysaccharides were measured with a TA.Touch texture analyzer (Shanghai Baosheng Industrial Development Co. Ltd., Shanghai, China). A P/36R probe was employed for the test, with pre-test, test, and post-test speeds of 2, 1, and 2 mm/s, respectively. The trigger force was fixed at 5 g, and the samples were compressed to 50% strain.

### 2.6. Intermolecular Forces Determination of Fish Balls

As reported previously [1], the fish balls (5 g) were homogenized with S1 (0.05 M NaCl), S2 (0.6 M NaCl), S3 (0.6 M NaCl + 1.5 M urea), or S4 (0.6 M NaCl + 8.0 M urea) at a ratio of 1:10 *m*/*v* and centrifuged at 8085× *g* for 20 min. The protein concentration in the supernatant was measured by the BCA kit (Xiamen Lanbolide Biotechnology Co., Ltd., Xiamen, China). The content of ionic bonds, hydrogen bonds, and hydrophobic interactions was calculated from the protein concentration difference between S2 and S1, S3 and S2, and S4 and S3, respectively.

### 2.7. Low-Field NMR Analysis of Fish Balls

As reported previously [16], fish ball samples incorporating different red seaweed polysaccharides were packed into cylindrical quartz tubes. The transverse relaxation time (T_2_) was determined using a low-field nuclear magnetic resonance (NMR) instrument (Model PQ001, Suzhou Niumag Corporation Co. Ltd., Suzhou, China). T_2_ measurements were performed with a Carr–Purcell–Meiboom–Gill (CPMG) pulse sequence, under the following parameters: 6 scans, 12,000 echoes, an interval of 1000 s between consecutive scans, and a time interval of 200 s between the 90° and 180° pulses. The acquired low-field NMR data were primarily analyzed via continuous distribution inversion and discrete exponential fitting.

### 2.8. Scanning Electron Microscopy (SEM) Observation of Fish Balls

As reported previously [17], fish ball samples incorporating different red seaweed polysaccharides were cut into 1 mm-thick slices. The slices were fixed in 2.5% glutaraldehyde at 4 °C for 12 h, rinsed with phosphate buffer for 15 min, and then dehydrated in a graded ethanol series (50%, 60%, 70%, 90%, and 100% *v*/*v*) for 15 min per concentration. After preparation, the samples were sputter-coated with gold and subsequently observed using a scanning electron microscope (Model S-4800, Hitachi Ltd., Tokyo, Japan).

### 2.9. Antioxidant Activity of Red Seaweed Polysaccharides

#### 2.9.1. DPPH· Free Radical Scavenging Capacity

As reported previously [13], a 2 mg/mL polysaccharide solution and a 0.1 mmol/L DPPH ethanol solution were prepared. Then, 0.5 mL of the polysaccharide solution was thoroughly mixed with 0.5 mL of the DPPH ethanol solution. The combination was allowed to react for 30 min under light-protected conditions. The absorbance at 517 nm was determined. The DPPH· scavenging rate was computed as:

(2) $$DPPH\cdot radical\;scavenging\;rate(\%)=(1-\frac{A_{1}}{A_{0}})\times 100\%$$

where A_1_ represents the absorbance of the sample solution, and A_0_ represents the absorbance of the blank solution prepared by substituting distilled water for the sample.

#### 2.9.2. ·OH Free Radical Scavenging Effect Capacity

As reported previously [18], a 2 mg/mL polysaccharide solution, a 9 mmol/L salicylic acid ethanol solution, a 9 mmol/L FeSO_4_ solution, and an 8.8 mmol/L H_2_O_2_ solution were prepared. The following solutions were sequentially added to a centrifuge tube: 0.1 mL polysaccharide solution, 0.1 mL salicylic acid ethanol solution, 0.1 mL FeSO_4_ solution, 0.6 mL distilled water, and 0.1 mL H_2_O_2_ solution. The absorbance at 510 nm was determined. The ·OH scavenging rate was computed as:

(3) $$\cdot OH\;radical\;scavenging\;rate(\%)=(1-\frac{A_{1}}{A_{0}})\times 100\%$$

where A_1_ represents the absorbance of the sample solution, and A_0_ represents the absorbance of the blank solution prepared by substituting distilled water for the sample.

#### 2.9.3. ABTS·^+^ Free Radical Scavenging Capacity

As reported previously [19], a 2 mg/mL polysaccharide solution, a 7 mmol/L ABTS solution, a 2.4 mmol/L K_2_S_2_O_8_ solution, and a 0.2 mol/L phosphate buffer (pH 7.0) were prepared. First, the ABTS·^+^ stock solution was prepared by mixing equal volumes of ABTS solution and K_2_S_2_O_8_ solution, and then incubated in the dark for 12 h. The ABTS·^+^ stock solution was then diluted with phosphate-buffered saline to achieve an absorbance value of 0.70 ± 0.02 at 734 nm. A 0.1 mL polysaccharide solution was mixed with 1 mL ABTS·^+^ working solution and incubated at 37 °C for 1 h. The absorbance at 734 nm was determined. The ABTS·^+^ scavenging rate was computed as:

(4) $${ABTS\cdot }^{+}\;free\;radical\;scavenging\;rate(\%)=(1-\frac{A_{1}}{A_{0}})\times 100\%$$

where A_1_ represents the absorbance of the sample solution, and A_0_ represents the absorbance of the blank solution prepared by substituting distilled water for the sample.

### 2.10. Antioxidant Capacity of Fish Balls and Their In Vitro Gastrointestinal Digesta

The fish balls were freeze-dried and ground into powder. The fish ball powder was weighed to prepare a 10 mg/mL fish ball solution. The antioxidant capacity determination method follows Section 2.9.

*In vitro* simulated gastrointestinal digestion was performed by the method of Minekus et al. [20] with minor modifications. Each 5 g of crushed fish balls was mixed with 5 mL simulated saliva (75 U/mL α-amylase, 0.75 mmol/L CaCl_2_, pH 7.0), pure water was used as the reference, and the reaction was carried out at 180 r/min and 37 °C for 5 min. The obtained oral digesta of 10 mL was mixed with 10 mL simulated gastric solution (2000 U/mL pepsin, 0.075 mM CaCl_2_, pH 3.0), incubated at 37 °C with shaking at 180 r/min for 180 min. Subsequently, 20 mL of simulated intestinal fluid (200 U/mL pancreatic α-amylase, 150 U/mL glucosidase, 0.3 mmol/L CaCl_2_, 10 mmol/L bile acid, pH 7.0) was added and then incubated at 37 °C with shaking at 180 r/min for 180 min. The digesta was inactivated at 100 °C for 10 min, centrifuged at 8085× *g* for 20 min, and the antioxidant capacity of its supernatant was determined as referenced in Section 2.9.

### 2.11. Statistical Analysis

Statistical analysis of experimental data was performed using IBM SPSS Statistics 26.0 software, with multiple comparisons conducted via the LSD method. All indicators underwent three parallel independent experiments (*n* = 3), with data presented as mean ± standard deviation (Mean ± SD). The significance level of *p* < 0.05 was set to indicate statistically significant differences.

## 3. Results and Discussion

### 3.1. Gel Strength of Fish Balls

**Figure 1:**
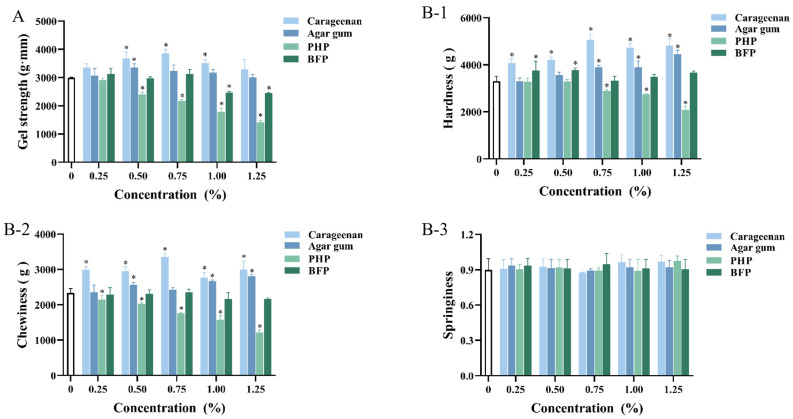
Effect of different red seaweed polysaccharides on the gel properties of fish balls: (**A**) Gel strength; (**B**) TPA; * represents significant difference compared to the control group (*p* < 0.05).

The ability of gel products to withstand deformation, fracture, and flow under external forces is known as gel strength, which is a crucial mechanical characteristic that reflects the stability and resilience of their network structure [16]. The gel strength of fish balls is shown in Figure 1A. When carrageenan and agar gum were added, the gel strength of fish balls showed an increasing and then decreasing trend. Compared with the blank groups, the highest gel strength of fish balls was observed when carrageenan was added at 0.75% and agar gum at 0.50%, respectively, which increased by about 28.84% and 12.08% (*p* < 0.05). The enhanced gel strength of fish balls is mainly ascribed to the water-absorbing and swelling behavior of carrageenan and agar gum under heating conditions. The swollen polysaccharides occupy the voids in the gel network and impose mechanical pressure on the protein structure [21]. However, the addition of lower amounts of PHP and BFP did not significantly affect the gel strength of fish balls, but significantly decreased the concentration of PHP by ≥0.50% and BFP by ≥1.00% as compared to that of the blank group (*p* < 0.05). Results indicated that adding too much PHP and BFP could prevent fish ball proteins from cross-linking and obstruct the development of an ordered network structure, which would reduce gel strength. This effect can be attributed to the dilution of myofibrillar proteins by crude polysaccharides, which compromises the structural integrity of fish balls [22]. Yang et al. [23] have found that the gel properties of surimi decrease when laver powder exceeds the critical concentration. According to Montero et al. [24], the kind and concentration of additional polysaccharides have a direct impact on the textural properties of surimi products. Similarly, the gel strength of fish balls was correlated with the type and concentration of red seaweed polysaccharides.

### 3.2. TPA of Fish Balls

The influence of red seaweed polysaccharides on TPA including hardness, chewiness, and springiness of fish balls is presented in Figure 1B. At the same concentration, the hardness and chewiness of fish balls containing carrageenan were higher than the other samples and was highest at the concentration of 0.75%, which was consistent with its highest gel strength value (Figure 1A). Cao et al. [11] have reported that carrageenan enhances the hardness and chewiness of frankfurters, consistent with the findings of this study. Agar gum at 1.00% and 1.25% was able to significantly improve both hardness and chewiness (*p* < 0.05). This is primarily attributed to the addition of carrageenan and agar, which enhances the viscosity of the surimi products while promoting the formation of hydrogen bonds between carrageenan and proteins. This facilitates the development of a more compact gel network structure within the fish balls.

Compared to carrageenan and agar gum, low concentrations of PHP (0.25%–0.50%) had no significant effect on fish ball hardness (*p* ≥ 0.05). Fish balls’ hardness decreased significantly (*p* < 0.05) when PHP was added at concentrations of 0.75%–1.25%, consistent with the trend of reduced gel strength shown in Figure 1A. BFP exhibited a positive influence on fish ball hardness only at 0.25%–0.50%. Alipour et al. [25] have added sulfated polysaccharides from green alga *Ulva intestinalis* to surimi, finding that its firmness and chewiness reduced when the concentration exceeded 0.5 g/100 g. This is mostly because seaweed polysaccharides only function as a filler and interfere with the production of the gel matrix of fish balls since they cannot interact with the myofibrillar proteins to form a structured system. It has been shown that carrageenan may be gel in surimi and dispersed in a protein matrix, affecting the gel structure [24]. Thus, it was hypothesized that carrageenan and agar can swell physically by absorbing water to fill the protein matrix of fish balls. In contrast, flocculent PHP tends to aggregate and entangle under conditions of insufficient water; this not only occupies a large space but also fails to form an ordered structure with proteins, ultimately resulting in a poorly developed protein gel network.

However, red seaweed polysaccharides did not have a significant effect on the springiness of fish balls (*p* ≥ 0.05). Chen et al. [1] have also reported that none of the used additives (e.g., curdlan, xanthan gum, carrageenan, and gelatin) significantly affected the springiness of surimi products. In summary, the different structure, physical properties, type, and concentration of red seaweed polysaccharides have an important effect on the hardness and chewiness of fish balls, among which carrageenan and agar gum can improve the gel quality of fish balls.

### 3.3. Molecular Forces of Fish Balls

In the subsequent study, fish ball samples supplemented with 0.25%, 0.50%, and 1.00% (*w*/*w*) red seaweed polysaccharides were used as test subjects. The effects of red seaweed polysaccharides on the gel properties of fish balls were explored by analyzing the molecular forces, water distribution, and protein structure of the samples, with the aim of clarifying the relevant regulatory mechanism.

**Figure 2:**
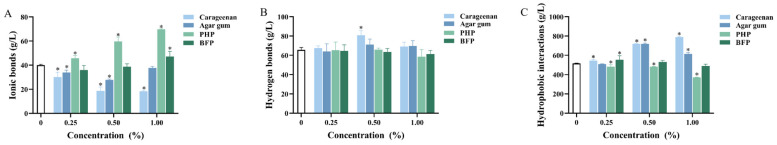
Molecular forces of fish balls; (**A**) Ionic bonds; (**B**) Hydrogen bonds; (**C**) Hydrophobic interactions; * represents significant difference compared to the control group (*p* < 0.05).

Molecular forces such as the ionic bonds, hydrogen bonds, and hydrophobic interactions of fish balls with red seaweed polysaccharides are shown in Figure 2. With increasing concentrations of carrageenan and agar gum, the ionic bonds in fish balls weakened, suggesting improved gel properties. This is due to the fact that during surimi gel formation, the degree of protein cross-linking usually rises when ionic connections are broken. However, when excessive amounts of hydrocolloids are added to surimi gels, pH variations may alter the charge distribution of amino acid residues. As a result, ionic bonds somewhat increase and gel strength subsequently decreases [26]. In addition, an increase in the concentration of PHP and BFP, especially PHP, significantly increased ionic bond content (*p* < 0.05) and was negatively correlated with gel strength (Figure 1A). Previous studies have reported that the gel strength of surimi is negatively correlated with the ionic bond content [1,27], which is in agreement with our results.

Regarding hydrogen bonds, all red seaweed polysaccharides except carrageenan had no significant effect on the hydrogen bond content of fish balls (*p* ≥ 0.05). When compared with the blank group, the addition of 0.50% carrageenan significantly increased the hydrogen bond content of the fish balls by 23.05%. Chen et al. [1] have found that added carrageenan (1–2 g/kg) to silver fish surimi could increase the hydrogen bond content. This is primarily because carrageenan enhances the water-holding capacity of fish paste systems by forming hydrogen bonds with free water, thereby improving their gel properties. However, ionic bonds and hydrogen bonds are not the dominant forces maintaining the gel network structure of fish balls; hydrophobic interactions are the primary molecular forces stabilizing the gel network, since the main component of fish balls is myofibrillar protein, whose molecular structure contains numerous hydrophobic amino acid residues. During heating, protein denaturation occurs, which unfolds the helical structure of protein molecules, exposing hydrophobic groups [28]. These hydrophobic groups aggregate with each other, forming a stable three-dimensional network structure. The results indicate that hydrophobic interactions account for the highest percentage of chemical molecular forces in fish balls to stabilize the fish ball gel network, as reported previously [1,29]. This phenomenon is also highly consistent with our results. The hydrophobic interactions of fish balls were significantly increased with the addition of 0.25%, 0.50%, and 1.00% carrageenan, as well as 0.50%–1.00% agar gum. This increase, in turn, enhanced the gel strength of the fish balls. However, the addition of PHP significantly decreased the hydrophobic interactions of the fish balls (*p* < 0.05). This may be attributed to the water-absorbing property of PHP, which causes it to occupy most of the water in the complex system of fish balls. This, in turn, hinders the binding of proteins to water molecules, ultimately resulting in poorer gel strength, hardness, and chewiness of the final fish ball products. In addition, 0.25% BFP significantly increased the hydrophobic interaction of fish balls (*p* < 0.05), but 0.50%–1.00% BFP had no significant effect on hydrophobic interaction (*p* < 0.05). Overall, it can be seen that hydrophobic interaction was closely related to the gel strength and TPA of fish balls with red seaweed polysaccharides.

### 3.4. Low-Field NMR of Fish Balls

**Figure 3:**
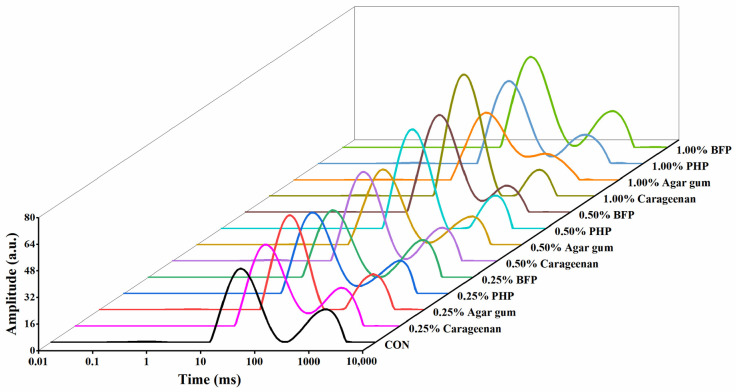
Low-field NMR of fish balls.

The effects of red seaweed polysaccharides on the types and distribution of water molecules in fish balls were analyzed using low-field NMR technology, as shown in Figure 3 and Table 1. The fish ball protein gel mainly included three types of water molecules: protein-associated water T_21_ (0.1–10 ms), immobilized water T_22_ (100–500 ms), and free water T_23_ (500–2000 ms), where mobility is enhanced in order [30]. Protein-associated water T_21_ is bound to protein molecules through covalent bonds, which is dependent on the protein gel itself [31]. The peak area of fish balls T_21_ was small, which did not exceed 0.90%, and decreased upon the addition of most red seaweed polysaccharides. The polysaccharide type and concentration had a significant effect on the relaxation times T_22_ and T_23_. The addition of 0.50%–1.00% carrageenan and agar gum increased the T_22_ ratio and decreased the T_23_ ratio when compared with the control samples, indicating that a portion of free water was converted into bound water and retained within the fish balls [32]. The T_22_ ratio increased when PHP and BFP were added to fish balls, although this effect was inconsistent with the fish ball gel strength trend. This could be explained by the tendency of PHP and BFP to self-wrap and aggregate under conditions of insufficient moisture [17]. The interactions between proteins and water molecules within the gel network are disrupted when the polysaccharides that have collected inside it bind with extra water molecules, thereby affecting the gel strength of fish balls.

### 3.5. SEM Images of Fish Balls

**Figure 4:**
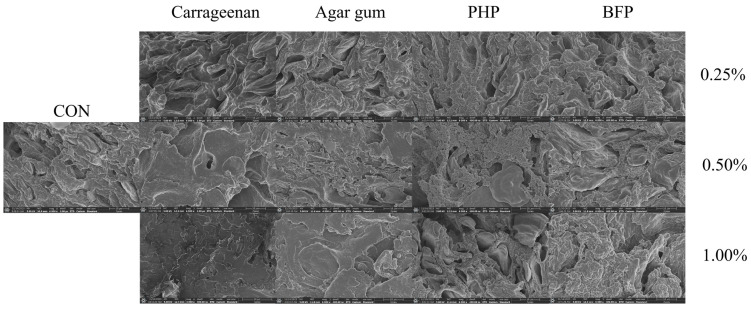
Microstructure of fish balls (6000×).

Scanning electron microscopy (SEM) is commonly employed to examine the microstructure of surimi gels, with surface roughness and pore size serving as key evaluation metrics for gel structure. This study employed SEM to observe the microstructure of fish balls (Figure 4). The fish balls in the CON group exhibited a loose network structure, with large gel pores and a dispersed structure. The network structure of the fish balls remained unchanged when 0.25% red seaweed polysaccharides was added. With the addition of 0.50% red seaweed polysaccharides, fish balls had a denser and flatter gel network structure with smaller pores, indicating that a more compact structure had formed. With the increase in the concentration of red seaweed polysaccharides, 1.00% carrageenan and agar gum significantly improved the structure of fish balls, which exhibited a more dense and flat structure. Chen et al. [1] have discovered that adding carrageenan to silver carp surimi gels may encourage the development of a more compact and ordered protein structure. This was mainly because carrageenan and agar gum were uniformly dispersed in the surimi gel, forming a gel to fill the voids of the surimi gel network structure at low temperatures, which had a supporting and reinforcing effect on the surimi protein structure. However, the gel structure of fish balls became rougher when 1.00% PHP and BFP were added, in particular, the addition of PHP caused the gel structure to form big, irregular aggregates and the pores to enlarge. This may be related to the solubilizing nature of polysaccharides, as the powdered particles of PHP and BFP were larger and irregular compared to carrageenan and agar gum. When making fish balls, the red seaweed polysaccharides were directly fed without cold water pre-dissolution of PHP and BFP, and the poor solubility of the two resulted in the fish balls also containing some incompletely dissolved polysaccharide particles, which prevented the gel network structure from forming. In conclusion, the gel network structure of fish balls could be positively impacted by the addition of 0.50% and 1.00% carrageenan and agar gum in the microstructure, and its macroscopic manifestation was the increase in gel strength, hardness, and chewiness of fish balls as discussed above, while 1.00% PHP had the opposite effect. Therefore, these polysaccharides might need a different incorporation method (e.g., pre-dissolution) to avoid negative effects on gel texture.

### 3.6. Free Radical Scavenging Ability of Red Seaweed Polysaccharides

Free radicals refer to molecules or ions bearing unpaired electrons; due to their highly reactive chemical nature, they readily react with nearby biomolecules, causing oxidative damage. Research indicates that excessive accumulation of free radicals is associated with various pathological processes, including aging, cancer development, and inflammatory responses [33]. Antioxidant activity has been evaluated using DPPH·, ABTS·^+^, and ·OH free radical scavenging capabilities. According to Table 2, PHP and BFP possessed a better DPPH· radical scavenging capacity of 46.02% and 28.52%, respectively, whereas carrageenan and agar gum had a weak DPPH· radical scavenging effect of only 6%–8%. The ABTS·^+^ and ·OH radical scavenging capacities of red seaweed polysaccharides showed similar results. Previous studies have demonstrated that the antioxidant activity of polysaccharides is closely related to their sulfate and uronic acid content; higher levels of these components correlate with stronger antioxidant activity [13]. Our previous results have indicated that BFP possesses higher sulfate and uronic acid content than PHP [34]. Consequently, BFP exhibits superior antioxidant activity. As shown in Tables S1 and S2, despite carrageenan possessing a high sulfate content (24.11%) and agar gum exhibiting a high uronic acid content (16.56%), their antioxidant activities are relatively weak. Sun et al. [35] have also reported that at concentrations of 0.5–2.0 mg/mL, carrageenan only exhibited a DPPH· radical scavenging rate of approximately 10%. Thus, the poorer antioxidant capacity of carrageenan and agar gum may be attributed to their high molecular weight and compact structure, thus making the active functional groups in the molecule less likely to interact with free radicals [35].

### 3.7. Antioxidant Activity of Fish Balls

**Figure 5:**
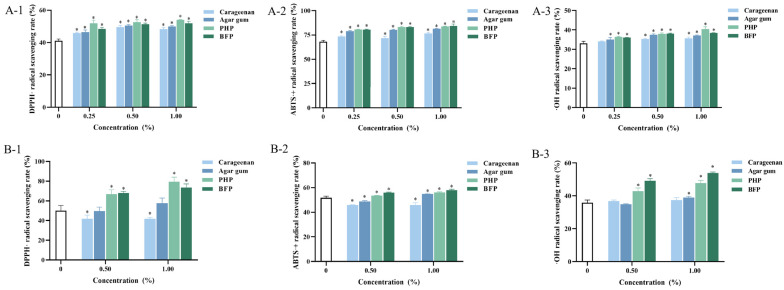
Antioxidant capacity: (**A**) fish balls with red seaweed polysaccharides; (**B**) in vitro digesta of fish balls; * represents significant difference compared to the control group (*p* < 0.05).

The antioxidant properties of fish balls were evaluated by determining the scavenging rate of DPPH·, ABTS·^+^, and ·OH radicals (Figure 5A). At a concentration of 1.00%, the DPPH· radical scavenging activities of fish balls containing carrageenan, agar gum, PHP, and BFP reached 48.28%, 49.95%, 54.02%, and 51.87%, respectively, all significantly higher than the control group (41.07%, *p* < 0.05). The ABTS·^+^ radical scavenging of fish balls was also increased by the addition of red seaweed polysaccharides (*p* < 0.05), and the best scavenging ability of 84.01% was found in fish balls with 1.00% BFP. The addition of 0.50% and 1.00% carrageenan significantly increased the ·OH radical scavenging ability of fish balls (*p* < 0.05). In general, these findings demonstrated that the antioxidant activity of fish balls was enhanced with increasing levels of red seaweed polysaccharides, and the optimal effect was observed at an addition level of 1.00%. Similarly, Zheng et al. [12] have incorporated fucoidan into fish balls and found that the fucoidan enhanced the DPPH·, ABTS·^+^, and ·OH radical scavenging capabilities of fish balls. Vikash et al. [36] have found that phenolic-rich green tea can enhance the gel properties and inhibit lipid and protein oxidation in surimi products. In this study, although carrageenan and agar gum were found to have no strong free radical scavenging capacity (Table 2), their addition still enhanced the antioxidant capacity of fish balls. This may be attributed to the enhancing effects of carrageenan and agar gum on the gel properties of fish balls. The denser gel structure can impede the contact between oxidizing agents such as free radicals and easily oxidized components within the fish balls, such as fats and proteins, thereby improving their antioxidant capacity [14]. In contrast, the textural and gel characteristics of fish balls were negatively impacted by the addition of PHP and BFP. Therefore, rather than being related to the changed gel characteristics, the increased antioxidant capacity of the fish balls is probably due to the significant antioxidant activity of PHP and BFP (Table 2). In conclusion, the incorporation of red seaweed polysaccharides can effectively inhibit free radical oxidation, which in turn delays protein oxidation and enhances the nutritious value of fish balls.

### 3.8. Antioxidant Activity of Fish Balls After Simulated Digestion In Vitro

With the addition of 0.50% and 1.00% red seaweed polysaccharides, fish balls had better free radical scavenging ability; thus, these fish balls were selected for *in vitro* oral gastrointestinal digestion. The antioxidant activities of the fish balls after simulated digestion were evaluated, with the results displayed in Figure 5B. After digestion, the DPPH·, ABTS·^+^, and ·OH radical scavenging rates of the control group were 50.17%, 51.72%, and 35.79%, respectively. The addition of 0.50% and 1.00% carrageenan had no significant effect on the ·OH radical scavenging capacity of the fish ball digestive solution, but the DPPH· and ABTS·^+^ radical scavenging rates were significantly decreased (*p* < 0.05), and 0.50% agar gum significantly reduced the ABTS·^+^ radical scavenging rate (*p* < 0.05). This is may be caused by the fact that the reduction in starch hydrolysis and protein digestion rates associated with the increased gel strength of fish balls, and the corresponding digestive solution will have lower contents of protein and reducing sugars, leading to the lower antioxidant activity of the digesta of fish ball. The 1.00% agar gum increased the scavenging rate of ABTS·^+^ and ·OH radicals in the digesta of fish balls (*p* < 0.05). Additionally, the radical scavenging capacity of digesta of fish balls increased with rising concentrations of PHP and BFP (*p* < 0.05), reaching its optimal level at 1.00% addition. Oh et al. [37] have investigated the antioxidant activity of *Paralichthys olivaceus* surimi digest (POSD) and found that POSD possessed significant DPPH· radical scavenging activity and was able to inhibit AAPH-stimulated apoptosis and necroptosis in Vero cells. Zeng et al. [38] have found that after simulated gastrointestinal digestion of LBP polysaccharides, the amount of monosaccharides increased significantly, and the total protein and phenolic content increased, resulting in improved ABTS·^+^ radical scavenging activity. Feng et al. [39] have demonstrated that polysaccharides from Chinese yam after gastrointestinal digestion increased its DPPH· radical scavenging activity due to the glycosidic bond rupture and the increase in the content of reducing sugars. Therefore, it was hypothesized that the better antioxidant capacity of fish balls with PHP and BFP after digestion is not only due to the increased protein digesta and reducing sugars but is also correlated with the relatively stronger antioxidant capacity of PHP and BFP (Table 2). Furthermore, existing studies indicate that peptides or amino acids present after digestion are one of the primary reasons for the high antioxidant activity of surimi products [36,40]. As previously reported, the gel structure of fish balls supplemented with PHP and BFP is more loosely packed, promoting the digestion of protein and the release of amino acids, which may increase the content of antioxidant peptides and make them more accessible [34]. This results in higher antioxidant activity in the digestive products.

## 4. Conclusions

This study explored the effects of red seaweed polysaccharides on the gel properties and *in vitro* antioxidant activity of fish balls (both before and after digestion). The findings exhibited that adding carrageenan and agar enhanced the gel strength, hardness, and chewiness of fish balls, with the optimal concentrations being 0.75% for carrageenan and 0.50% for agar gum, respectively. The addition of carrageenan and agar gum significantly increased hydrophobic interactions, elevated the proportion of immobilized water, and decreased free water content. This facilitated the binding between water molecules and proteins, exerting a positive effect on the gel network structure of fish balls. In contrast, excessive amounts of PHP and BFP inhibited the cross-linking of surimi proteins, thereby reducing gel strength, which may be caused by the suppressed intermolecular forces and the formation of ordered structures in fish balls with high concentrations of PHP and BFP. In terms of antioxidant activity, incorporating red seaweed polysaccharides improved the free radical scavenging capacity of fish balls. Specifically, after digestion, fish balls supplemented with 0.50% or 1.00% PHP and BFP exhibited superior free radical scavenging activity. These findings suggest that carrageenan and agar are suitable for producing high-quality and well-gelling fish balls, while PHP and BFP can be used to develop value-added products with enhanced antioxidant properties, provided their negative textural effects are mitigated.

## Figures and Tables

**Table 1:** Water distribution of fish balls.

	Concentration (%)	Area			Proportion (%)		
		T_21_	T_22_	T_23_	P_21_	P_22_	P_23_
CON	/	7.853	911.291	366.288	0.611	70.894	28.495
Carrageenan	0.25%	0.216	1094.069	445.853	0.014	71.028	28.945
	0.50%	4.289	1113.823	362.003	0.289	75.123	24.416
	1.00%	0.027	1084.023	377.99	0.002	74.145	25.854
Agar gum	0.25	0.136	844.608	409.000	0.011	67.367	32.622
	0.50	13.191	1130.622	356.853	0.878	75.293	23.764
	1.00	5.598	1007.259	331.967	0.416	74.899	24.685
PHP	0.25	0.998	1191.469	294.785	0.067	80.099	19.818
	0.50	4.211	1307.421	274.567	0.265	82.375	17.299
	1.00	10.112	1480.844	220.097	0.591	86.494	12.855
BFP	0.25	5.567	1039.94	306.297	0.411	76.813	22.624
	0.50	9.404	1087.023	360.531	0.644	74.458	24.695
	1.00	0.096	1133.239	405.31	0.006	73.530	26.298

**Table 2:** Free radical scavenging ability of red seaweed polysaccharides at 2 mg/mL.

Red Seaweed Polysaccharide	DPPH· Radical Scavenging Rate (%)	ABTS·^+^ Radical Scavenging Rate (%)	·OH Radical Scavenging Rate (%)
Carrageenan	6.00 ± 0.61 ^d^	1.64 ± 0.44 ^c^	6.22 ± 1.58 ^c^
Agar gum	7.96 ± 0.24 ^c^	2.33 ± 0.37 ^c^	6.45 ± 1.43 ^c^
PHP	28.52 ± 0.37 ^b^	30.66 ± 0.53 ^b^	10.33 ± 0.40 ^b^
BFP	46.02 ± 0.24 ^a^	65.03 ± 0.37 ^a^	13.91 ± 0.58 ^a^

Results were presented as mean ± standard deviation. Different letters superscripted on the results indicate significant difference (*p* < 0.05) between each attribute tested.

## Data Availability

The raw data supporting the conclusions of this article will be made available by the authors (zmjfst@163.com; zdjiang@jmu.edu.cn) on request.

## Supplementary Materials

The following supporting information can be downloaded at: https://www.mdpi.com/article/10.3390/foods15061018/s1, Table S1: The chemical composition of carrageenan and agar gum; Table S2: The monosaccharide composition of carrageenan and agar gum.
