# Supplementary material for: Influence of Red Seaweed Polysaccharides on Gel Properties and In Vitro Antioxidants of Surimi Product Fish Balls
Source: Foods. 2026 Mar 13;15(6):1018. doi: 10.3390/foods15061018 (PMC13025484; doi:10.3390/foods15061018)
Supplement: Supplementary file 1 [file foods-15-01018-s001.zip › foods-4168675-supplementary.pdf]

Supplementary Material

Table S1 The chemical composition of carrageenan and agar gum.

Table S2 The monosaccharide composition of carrageenan and agar gum.

Table S1 The chemical composition of carrageenan and agar gum.

**Red seaweed polysaccharide**	**Total sugar (%)**	**Protein (%)**	**Sulfate (%)**	**Uronic acid (%)**	**3,6-AG (%)**
carrageenan	47.20±0.62^{b}	0^{a}	24.11±0.49^{a}	9.07±0.32^{b}	26.39±0.14^{b}
agar gum	72.52±1.00^{a}	0^{a}	3.24±0.18^{b}	16.56±0.14^{a}	28.42±0.27^{a}

Different lowercase letters of the same column represent significant difference, $p < 0.05$.

Table S2 The monosaccharide composition of carrageenan and agar gum.

**Red seaweed polysaccharide**	**Glc-A (%)**	**Gal-A (%)**	**Glc (%)**	**Gal (%)**	**Fuc (%)**
carrageenan	1.56±0.06^{a}	1.18±0.02^{a}	1.17±0.02^{b}	93.44±0.10^{a}	2.64±0.00^{b}
agar gum	1.51±0.03^{a}	4.00±3.20^{a}	6.00±3.48^{a}	85.42±0.31^{b}	3.07±0.01^{a}

Different lowercase letters of the same column represent significant difference, $p < 0.05$.
